# Influence of *Bacillus subtilis* and *Trichoderma harzianum* on Penthiopyrad Degradation under Laboratory and Field Studies

**DOI:** 10.3390/molecules25061421

**Published:** 2020-03-20

**Authors:** Magdalena Podbielska, Małgorzata Kus-Liśkiewicz, Bartosz Jagusztyn, Bartosz Piechowicz, Stanisław Sadło, Magdalena Słowik-Borowiec, Magdalena Twarużek, Ewa Szpyrka

**Affiliations:** 1 Department of Biotechnology, Institute of Biology and Biotechnology, University of Rzeszów, Pigonia 1, 35-310 Rzeszow, Poland; mkus@univ.rzeszow.pl (M.K.-L.); bartoszjagusztyn@wp.pl (B.J.); m.slowik_borowiec@interia.pl (M.S.-B.); ewaszpyrka@interia.pl (E.S.); 2 Department of Animal Physiology and Reproduction, Institute of Biology and Biotechnology, University of Rzeszów, Pigonia 1, 35-310 Rzeszow, Poland; bpiechow@poczta.onet.pl; 3 Department of Ecotoxicology, Faculty of Biotechnology, University of Rzeszów, Pigonia 1, 35-310 Rzeszow, Poland; s.sadlo@poczta.onet.pl; 4 Department of Physiology and Toxicology, Institute of Experimental Biology, Faculty of Natural Sciences, Kazimierz Wielki University, Chodkiewicza 30, 85-064 Bydgoszcz, Poland; twarmag@ukw.edu.pl

**Keywords:** penthiopyrad, degradation, microorganisms, apples, consumer exposure

## Abstract

In plant protection, biological preparations are used alternately with chemical pesticides. The applied microorganism can influence the concentration of chemical substances. Laboratory and field studies were conducted to assess the influence of *Bacillus subtilis* and *Trichoderma harzianum* on the penthiopyrad concentration. In laboratory studies, the effectiveness of penthiopyrad degradation by *B. subtilis* was approximately 5% during 14 days of the experiment. For penthiopyrad treated with *T. harzianum* strains, the degradation effectiveness ranged from 34.2% on Day 3 to 56.9% on Day 14. In experiments testing the effects of mixed culture of microorganisms, the effectiveness of penthiopyrad degradation ranged from 23.7% on Day 3 to 29.1% on Day 14. After treatment of apple trees of Gala and Golden Delicious varieties with a biological preparation, a maximum degradation of penthiopyrad of 20% was found in both varieties. Samples of apples were prepared by the quick, easy, cheap, effective, rugged and safe (QuEChERS) method, and penthiopyrad was analyzed by gas chromatography with a mass detector. A determined value of the chronic exposure to penthiopirad was 1.02% of the acceptable daily intake, both for children and for adults. The acute exposure amounted to 7.2% and 1.9% of the acute reference dose for children and adults, respectively. These values were considered to be acceptable and not threatening to health.

## 1. Introduction

Pesticides are considered a vital component of farming, as they play a major role in maintaining high agricultural productivity [1]. Pesticides are commonly used to protect apple trees against diseases and pests. In consequence, their residues are present in fruit and may pose a risk of consumer exposure. In orchards managed under a conventional system, up to a dozen treatments are performed during the season to protect fruit against diseases of fungal origin [2]. To minimize crop losses caused by fungal pathogens, treatments with fungicide preparations from demethylation inhibitors (DMI), succinate dehydrogenase inhibitors (SDHI), and quinone outside inhibitors (QoI) groups are necessary [3]. In recent years, a second generation of SDHI fungicides has been introduced into the market, effective against diseases affecting cereal, fruit, and vegetable crops [4]. Penthiopyrad (IUPAC name: (*RS*)-*N*-[2-(1,3-dimethylbutyl)-3-thienyl]-1-methyl-3-(trifluoromethyl)pyrazole-4-carboxamide), belonging to SDHI, is a carboxamide fungicide used to control a broad spectrum of diseases in a large variety of crops: cereals (including wheat), potatoes, sugar beet, turf, legumes, vegetables, soybean, and apples [5]. SDHI fungicides inhibit fungal respiration by binding to the mitochondrial respiratory complex II [6]. In Poland, penthiopyrad is an active substance in Fontelis 200 SC and Orlian 200 SC, plant protection products recommended for apple protection [7].

The constant use of pesticides leads to a risk of cancer (in children and adults) [8], reproductive system disorders [9], birth defects [10], and diseases of the central nervous system such as Alzheimer’s or Parkinson’s [11]. Therefore, tools and methods are developed for determining relevant chemicals. Furthermore, efficient management practices are established, implementing regulations for monitoring the quality and distribution of vegetables and fruit, and ensuring their pesticide content is below maximum residue limits [12]. Methods for sample preparation and instrumental techniques for residue quantification are of great importance, because pesticide residues in samples are determined at a very low level (ppm, ppb). Pesticides are mainly determined by gas (GC) and liquid (LC) chromatography coupled with mass spectrometry [13]. Nowadays, the quick, easy, cheap, effective, rugged and safe (QuEChERS) method is very popular for preparation of samples of different matrices [14].

The growing problems associated with the use of pesticides in agriculture (toxic effects on human health and the environment, the development of resistance in plant pathogens and pests, etc.) are accompanied by an increasing interest in the use of biological methods, including microorganisms, to improve plant health and productivity, and ensure human safety and environmental protection [15,16] Microbiological preparations are used to protect plants and stimulate their growth and yield capacity. They are also used in bioremediation processes to eliminate problems associated with the use of chemical fertilizers, pesticides and polycyclic aromatic hydrocarbons (PAHs) [17,18]. Currently, new biotechnological methods for decomposing plant protection products are being sought in environmental biotechnology. Preparations containing microorganisms, used as mineral fertilizers and/or preparations for plant protection, are available on the market. It is recommended to use such preparations alternately with chemical preparations, to improve the quality and safety of produced food [19].

A review of the literature indicates a great interest in the use of bacteria and fungi for the degradation of active substances of pesticides. *Bacillus* spp. bacteria are used for the production of commercial preparations, including enzymes, insecticides, antibiotics, and vitamins, as well as other metabolites (hyaluronic acid) [20,21]. *Bacillus* spp. also degrade pesticides, usually insecticides including chlorpyrifos [22], aldrin, dieldrin, DDT [23,24,25] acibenzolar-S-methyl [26], diazinon [27], endosulfan [28], parathion-methyl [29], metribuzin [30], malation [31], cypermethrin [32], and quinalphos [33].

*Trichoderma* spp. are free-living fungi that are common in soil and root ecosystems. They are opportunistic plant symbionts, as well as parasites of other fungi. Fungi of the genus *Trichoderma* spp. inhibit and/or break down pectinases and other enzymes of fungal plant pathogens, such as *Botrytis cinerea* Pers., Fr. [34]. They produce or release various compounds, inducing those involved in local or systemic immune responses, such as lytic and proteolytic enzymes, as well as metabolites that can be used as biological fungicides to combat plant diseases caused by pathogenic fungi [35]. Formulations degraded by fungi of the genus *Trichoderma* spp. include chlorpyrifos [36,37], endosulfan and parathion-methyl [38], and carbendazim [39].

Available reports mainly concern the degradation of various active substances in laboratory conditions, with some of them focusing on identification of microorganisms isolated from soil and testing their ability to degrade selected active substances of pesticides.

The aim of the study was: (1) to check the efficiency of penthiopyrad biodegradation by reference strains of *Bacillus subtilis* and *Trichoderma harzianum* fungi and a mixed culture of microorganisms in laboratory conditions; (2) to check whether the use of biological preparations recommended in the Integrated Plant Protection Programme affects the degradation of penthiopyrad in field experiments. Furthermore, no data are available in the literature concerning the degradation of penthiopyrad residues in agricultural products, since it is a relatively new fungicide. Therefore, the additional aim was to determine its residue levels under field conditions, and establish dissipation kinetics in ripe apple fruit after foliar application of Fontelis 200 SC on apple trees of Gala and Golden Delicious varieties within 21 days before harvest. After the tests, the consumer exposure to residue intake associated with the consumption of apples by children and adults was also estimated.

## 2. Results

The study in laboratory conditions was conducted to check whether penthiopyrad degradation is affected by *B. subtilis* PCM 486 and *T. harzianum* KKP 534 strains. Additionally, laboratory studies focused on the viability/metabolic activity of *B. subtilis* cells and determined the minimum inhibitory concentration (MIC). In the next step, it was checked whether the treatment of apple orchards with a biological preparation changes a concentration of penthiopyrad applied on apple plants three weeks before fruit harvest.

### 2.1. Studies on Metabolic Activity of B. subtilis Cells

During the studies on the penthiopyrad degradation by *B. subtilis*, cell metabolic activity were conducted over 14 days. The bacterial cells were incubated in nutrient broth (NB), and an increase in the cellular metabolic activity was observed on Day 3, followed by a decrease from Day 5 onwards. This may reflect a depletion of nutrients in the medium (Supplementary Figure S1).

### 2.2. Determination of MIC of Penthiopyrad for B. subtilis and a Reference Fungus S. Cerevisiae

**Figure 1:**
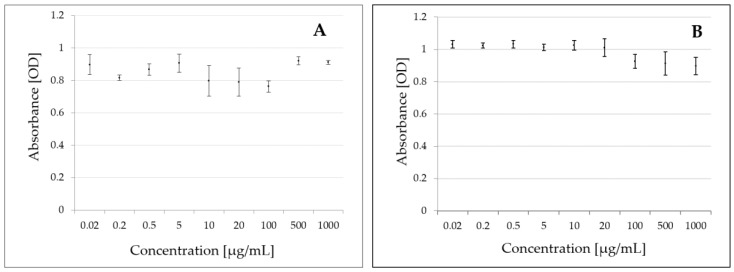
Inhibition of *B. subtilis* (**A**) and *S. cerevisiae* (**B**) growth after 24 h exposure to penthiopyrad.

Fungicides have a broad spectrum of effects on fungi, but there are no data on the effect of these substances on bacteria, such as *B. subtilis*. Therefore, a test was conducted to determine whether penthiopyrad inhibited the growth of reference fungal strains and of bacteria. Penthiopyrad concentrations selected for the analysis ranged from 0.02 to 1000 µg/mL. After the exposure of *B. subtilis* and of the reference fungi *S. cerevisiae* SP4 to penthiopyrad, no inhibition of bacterial and fungal growth was observed in the tested concentration range (Figure 1A,B).

### 2.3. Degradation of Penthiopyrad by B. subtilis, T. harzianum, and a Mixed Culture of B. subtilis and T. harzianum in Laboratory Conditions

**Figure 2:**
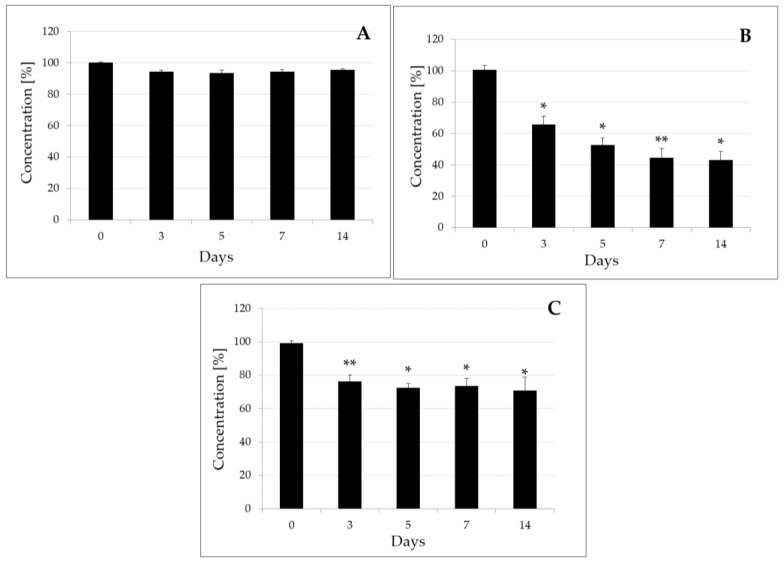
Concentration [%] of penthiopyrad treated with *B. subtilis* (**A**), *T. harzianum* (**B**) and a mixed culture of *B. subtilis* and *T. harzianum* (**C**).

The experiments tested the effects of *B. subtilis* and *T. harzianum* strains, and of a mixed culture of *B. subtilis* and *T. harzianum* on the degradation of penthiopyrad, the active substance of Fontelis 200 SC. During the experiment, samples were taken under sterile conditions on days 0, 3, 5, 7, and 14. In experiments with *B. subtilis*, on day zero the penthiopyrad concentration was 114.6 ± 5.1 µg/mL in control samples, and 114.8 ± 5.7 µg/mL in study samples subjected to bacterial degradation. Subsequently, the penthiopyrad concentration ranged from 104.0 ± 4.2 µg/mL to 112.5 ± 4.6 µg/mL in the control samples, and from 98.4 ± 4.8 µg/mL to 99.5 ± 3.5 µg/mL in the samples treated with bacteria (Table 1). The degradation of penthiopyrad was of up to 6.6% (Figure 2A).

In experiments with *T. harzianum* strains, the determined initial concentration of penthiopyrad was 106.0 ± 2.5 µg/mL in control samples and 106.7 ± 5.4 µg/mL in samples subjected to fungal degradation. Subsequently, the determined penthiopyrad concentration was in a range from 105.0 ± 2.8 µg/mL on Day 3 to 99.4 ± 5.1 µg/mL on Day 14 in the control samples, and from 69.1 ± 7.6 µg/mL on Day 3 to 42.9 ± 7.6 µg/mL on Day 14 in the degraded samples (Table 1). The effectiveness of penthiopyrad degradation ranged from 34.2% (Day 3) to 56.9% (Day 14) (Figure 2B).

In experiments testing the effect of the mixed culture of *B. subtilis* and *T. harzianum* on the penthiopyrad degradation, on day zero the penthiopyrad concentration was 110.6 ± 2.4 µg/mL in control samples and 109.8 ± 4.3 µg/mL in samples subjected to microbial degradation. During the experiment, the penthiopyrad concentration ranged from 100.8 ± 6.0 µg/mL to 109.3 ± 5.7 µg/mL in the control samples, and from 71.5 ± 3.5 µg/mL to 80.3 ± 2.1 µg/mL in samples degraded by microorganisms (Table 1). The penthiopyrad degradation was in the range of 23.7%–29.1% (Figure 2C).

### 2.4. Field Experiments

A day after the treatment with the biological preparation, no changes in penthiopyrad concentration in apples were observed in relation to the control samples, and its residues were at the level of 0.102 ± 0.018 mg/kg (control samples: 0.105 ± 0.036 mg/kg) (Table 2).

**Figure 3:**
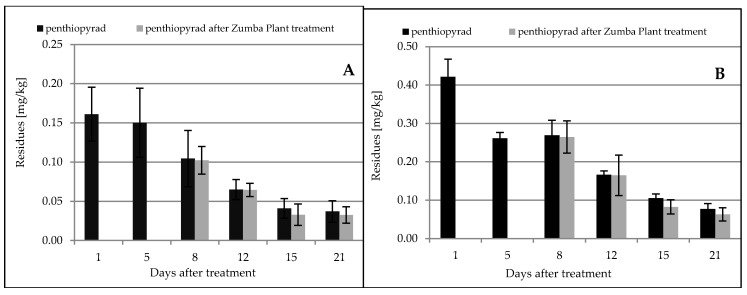
Penthiopyrad residues after application of formulations: Fontelis 200 SC on 8/16/2017 (**A**) and 9/4/2018 (**B**), and Zumba plant^®^ on 8/23/2017 (**A**) and 9/11/2018 (**B**).

On the second sampling date, the residues were at the same level as in the control samples. On the third and the fourth sampling dates after application of the biological preparation, a reduction in the penthiopyrad concentration by 20% and 14%, respectively, was observed in comparison to the control samples (Figure 3A).

After the application of the biological preparation Zumba Plant^®^ in the Golden Delicious variety, penthiopyrad residues were at the level of 0.265 ± 0.042 mg/kg, and were lower by 1.5% versus the control samples (Table 2). Similarly, on the next sampling date, the residues were lower by 0.6% vs. the control samples (Figure 3B). On September 19, residues reached 0.083 ± 0.018 mg/kg and were lower by 21% when compared to the controls (Table 2, Figure 3B). On the last day of the experiment, penthiopyrad residues were at 0.063 ± 0.017 mg/kg, and lower by 19% when compared to control samples (Table 2, Figure 3B).

### 2.5. Penthiopyrad Dissipation Kinetics

Changes in the average residue levels in apples were fitted to first-order kinetics using the following exponential equation:

P_t_ = P_0_ × e^−kt^ (1)

where P_0_ is the initial residue concentration (for t = 0), and k (day^−1^) is a constant rate of those changes. The constant k was used to estimate a half-time value: t_1/2_ = ln2/k.

**Figure 4:**
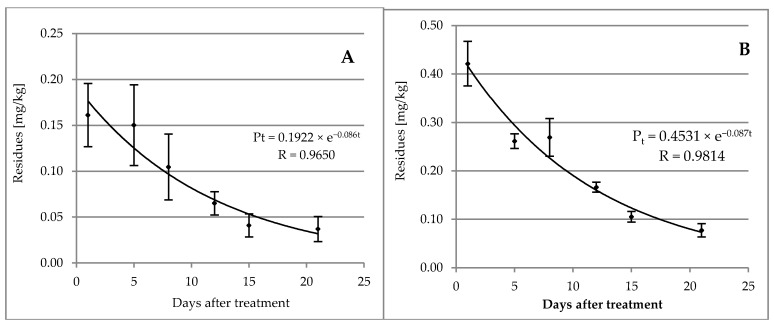
Penthiopyrad dissipation kinetics after treatment with Fontelis 200 SC in the Gala variety on 8/16/2017 (**A**), and in the Golden Delicious variety on 9/4/2018 (**B**).

In the Gala variety, on the next day after the treatment with the chemical preparation penthiopyrad residue levels in the fruit reached 0.161 ± 0.034 mg/kg (Table 2). The dissipation kinetics of penthiopyrad were determined according to the exponential equation: P_t_ = 0.1922 × e^−0.086t^ (R = 0.9650), and its half-life was 8 days (Figure 4A).

After 21 days from the treatment, on the harvest day, the penthiopyrad content in apples was 0.037 ± 0.014 mg/kg (Table 2).

In the Golden Delicious variety, on the first day after the treatment with Fontelis 200 SC, penthiopyrad residues in the apple samples were at a level of 0.421 ± 0.046 mg/kg. They decreased to a level of 0.078 ± 021 mg/kg after 21 days of the experiment (Table 2). On the basis of the obtained results, the dissipation kinetics for the Golden Delicious variety were determined and described by the exponential equation: P_t_ = 0.4531 × e^−0.087t^ (R = 0.9814) (Figure 4B). Using that equation, its half-life (t_1/2_) of eight days was also determined.

### 2.6. Mycotoxins in Apple Samples

Patulin, trichothecenes: diacetoxyscirpenol deoxynivalenol, HT-2 toxin, T-2 toxin, nivalenol, and zearalenone were not found in the tested samples.

### 2.7. Estimation of Consumer Exposure

The risk to the health of children and adults posed by the long-term exposure, as estimated on the basis of the level of determined residues in the analyzed apple samples, indicates that the pesticide intake does not exceed the safe ADI (acceptable daily intake) level. For an average consumer, IEDI (International Estimated Daily Intake) was 1.02 (expressed as % of ADI). The acute exposure of children and adults to pesticide residues in apples did not exceed the ARfD (acute reference Dose) value. IESTI (international estimate of short-term intake) was 0.0539 mg/kg bw/day (7.2% ARfD) for children, and 0.0140 mg/kg bw/day (1.9% ARfD) for adults.

### 2.8. Method Validation

Validation was performed using apples and NB and Potato Dextrose Broth (PDB), to determine linearity, recovery, and precision. All analyses were performed with a reference to a standard mixture prepared in blank matrix. Precision was determined during recovery studies (five repetitions) and expressed as RSD. The average recoveries, RSDs, and linearity for penthiopyrad met the EU criteria for the pesticide analysis described in SANTE documents [40]. Detailed data are given in Supplementary Tables S1–S2.

## 3. Discussion

In cell viability/metabolic activity studies for *B. subtilis* in the presence of penthiopyrad, cell survival was observed (Supplementary Figure S1). MIC tests showed no inhibition of *B. subtilis* and of a reference fungi *S. cerevisiae* growth by penthiopyrad in the concentration range of 0.02–1000 µg/mL. (Figure 1A,B). In the laboratory studies, penthiopyrad degradation was up to 6.6% for *B. subtilis* (Figure 2A), and 34.2%–56.9% for *T. harzianum* strains (Figure 2B). When the mixed culture of microorganisms was used, the effectiveness of penthiopyrad degradation was lower than when using the bacterial and fungal strains alone, and was in the range of 23.7%–29.1% (Figure 2C). The obtained results may indicate that the microorganisms had an inhibitory effect on each other [41].

In scientific literature no data is available on the penthiopyrad degradation by microorganisms under laboratory or field conditions. The majority of publications concerning fungicides undergoing degradation by *Bacillus* spp. and *Trichoderma* spp. strains focus on substances from the benzimidazole group, which mechanism of action is different than that of the fungicides discussed in this paper. Zhang et al. [42] report that the effectiveness of carbendazim degradation by *B. pumilus* NY97-1 at concentrations of 10, 30, 50, 100, and 300 mg/L was 42.44%, 48.97%, 77.19%, 78.66%, and 90.07%, respectively. Salunkhe et al. [43] report that the degradation of carbendazim by four *B. subtilis* strains, TL-171, TS-204, DR-39 and CS-126, was within the range of 75.7%–95.2%. A degradation effectiveness of 73.2% was also observed by Panda et al. [44] for *B. licheniformis* JTC-3 bacteria. Sharma et al. [39] determined the biodegradation effectiveness of carbendazim as 85% for *T. harzianum,* 47% for *T. viride*, and 21% for *T. atroviride*. Cycoń et al. [45] conducted a study on the degradation of thiophanate methyl by *Bacillus* sp. TDS-2 strains, determining its effectiveness at a level of 77%. Youness et al. [46] report that gram-positive bacteria degrade tebuconazole by 30% in the case of *B. cereus* 2B9 and *Bacillus* sp. IB13, and by 50% in the case of *Bacillus* sp. 29B3 and *Bacillus* sp. 3B6. The degradation of tebuconazole is also influenced by another microorganism, *Nocardia asteroides* LAB 911 (35%), while gram-negative bacteria *Pseudomonas* sp. C12B ATCC 43648 degrade it by 50%. Tebuconazole is not degraded by fungi from the genus *Aspergillus niger* ATCC 9142, *Aureobasidium pullulans G*, or *Geotrichum candidum* CBS 14488, while yeasts *Candida parapsilosis* ATCC 2046 degrade tebuconazole by 16%. In the case of degradation by bacteria *Serratia marcescens* B1 following the treatment with tebuconazole at a concentration of 200 mg/L, the degradation effectiveness was 94.05%. When a higher initial concentration of 300 mg/L was used, degradation effectiveness decreased with the increasing concentration of tebuconazole, reaching a level of 64.11% for 500 mg/L [47]. In studies conducted by Obanda et al. [48], tebuconazol was degraded by *T. harzianum* with the effectiveness of 68% in 21 days.

Treatments with Fontelis 200 SC at a dose of 0.75 L/ha, containing 200 g/L of the active substance (penthiopyrad) were conducted in the apple varieties: Gala, in Józefów nad Wisłą (2017), and Golden Delicious, in Rzeszów (2018). Fontelis 200 SC is a preparation of a contact, deep and local systemic action for preventive or interventional applications [49].

The initial penthiopyrad concentrations in apples of Gala and Golden Delicious varieties were 0.161 ± 0.034 mg/kg and 0.421 ± 0.046 mg/kg, respectively (Table 2). A temperature could also contribute to these differences. In the orchard in Józefów nad Wisłą, temperatures were about 5 °C higher, which could cause a faster dissipation rate and lower initial residue levels of penthiopyrad (Supplementary Figure S2A,B). Nevertheless, in the kinetic equations for the first order reactions, the initial concentration does not affect the half-life time. In both varieties, the half-life determined for penthiopyrad was eight days. The course of changes in penthiopyrad residue levels was described by kinetic equations: P_t_ = 0.1922 × e^−0.086t^ (R = 0.9650) and P_t_ = 0.4531 × e^−0.087t^ (R = 0.9814) in Gala and Golden Delicious varieties, respectively (Figure 4A,B).

After the biological treatments, the distribution of penthiopyrad residues in the fruit was similar in both orchards. On the first two sampling dates, no degradation of penthiopyrad by microorganisms was observed, while on Day 15 of the experiments, a 20% degradation was found in both varieties, and on the last day of the experiments the residue levels decreased by 14% and 19% in Gala and Golden Delicious varieties, respectively (Figure 3A,B). Weather conditions prevailing during the experiment (a temperature of 10–27 °C and no rainfall after application) affected the activity of microorganisms and the penthiopyrad degradation. The results obtained correlate with data obtained in laboratory studies on degradation by a mixture of bacteria and fungi. The conducted research indicates that weather conditions are an important factor influencing the possibility of using microorganisms for the penthiopyrad degradation. The results obtained in our previous studies, in which boscalid and pyraclostrobin were degraded by the biological preparation Zumba Plant^®^, indicate that the high effectiveness of decomposition is associated with weather conditions, because the temperature plays an important role in the development of antagonistic fungi, determining the fungal activity [50].

No data are available in the literature regarding the penthiopyrad disappearance in plants. Only the PPDB database reports that in melon its half-life is 3.1 days [5]. Further research is needed to determine the environmental fate of this active substance.

In our study, we focused on the determination of patulin, trichothecenes: diacetoxyscirpenol and deoxynivalenol, HT-2 toxin, T-2 toxin, nivalenol, and zearalenone, because the biological preparation used in the experiments contains fungi that can produce a number of metabolites. These mycotoxins were not found in the tested samples.

Determined values of the consumer exposure that did not exceed 100% of the ADI and ARfD values were considered to be acceptable and not threatening to health of children and adults.

## 4. Materials and Methods

### 4.1. Laboratory Trials

Three microbial strains were used: *Bacillus subtilis* PCM 486, *Saccharomyces cerevisiae* SP4 (both from the Culture Collection of University of Rzeszow, Poland), and *Trichoderma harzianum* KKP 534 (Institute of Agricultural and Food Industry, Poland). Tests were conducted under sterile static conditions, by incubation of *B. subtilis* and *T. harzianum* with the applied chemical preparation, Fontelis 200 SC (100 µg/mL). The tests were carried out in triplicate. Additionally, penthiopyrad concentrations inhibitory effect on *B. subtilis* and a reference fungus *S. cerevisiae* SP4, and cell viability of *B. subtilis* were evaluated.

#### 4.1.1. Determination of Concentration Inhibitory Effect on *B. subtilis* and a Reference Fungus *S. Cerevisiae*

A concentration of penthiopyrad inhibitory effect on *B. subtilis* and *S. cerevisiae* was examined by the MIC test. The MIC assay was done according to the standard protocols [51].

*B. subtilis* strain was inoculated into a tube with a NB containing meat extract (2 g/L), yeast extract (2 g/L), peptone (5 g/L), NaCl (4 g/L), and glucose (10 g/L) (BTL, Poland). The reference S. cerevisiae strain was inoculated into a tube containing glucose potato broth (PDB) with potato extract (4 g/L) and glucose (20 g/L) (BTL, Poland). Strains were incubated overnight at 37 °C and 30 °C for bacteria and fungi, respectively, with shaking at 150 rpm (Incubator MaxQ 6000, Thermo Fisher Scientific, Waltham, MA, USA). Bacteria and yeast cultures were diluted to match the optical density OD 600 ~ 0.1, which corresponds to 1.5 × 10^6^ and 5 × 10^5^, respectively. 100 µL of cells suspensions were distributed to the 96-well microtiter plate. The penthiopyrad standard (Supelco Inc., Bellefonte, PA, USA) was added to selected wells at a concentration ranging from 0.02 to 1000 µg/mL (prepared in methanol). The positive control contained the same amount of methanol. The negative control was the pure culture medium, and the positive control were strains of *B. subtilis* and *S. cerevisiae* not treated with penthiopyrad. All analyses were carried out in triplicate. The microtiter plates were incubated for 24 h at 30 °C and 37 °C, for bacteria and yeast strains, respectively. The absorbance was measured at 600 nm (Infinite M200, Tecan Group Ltd., Männedorf, Switzerland), at “zero” time and after 24 h. The lowest concentration of the tested nanosuspensios, which was transparent, was considered as the MIC.

#### 4.1.2. Degradation of Penthiopyrad by *B. subtilis*, *T. harzianum*, and a Mixed Culture of Bacteria and Fungi in Laboratory Conditions

*B. subtilis* strains and *T. harzianum* were inoculated into tubes containing sterile NB and sterile PDB, respectively, and incubated overnight at 37 °C for bacteria and 30 °C for fungi. Degradation tests were carried out in 100 mL Erlenmeyer flasks containing 20 mL of 10-fold diluted sterile NB and sterile PDB. For the mixed culture of microorganisms, the volume of broths in the flask was 10 mL of each. A solution of the chemical preparation Fontelis 200 SC was added to the Erlenmeyer flasks in a quantity sufficient to achieve a final concentration of penthiopyrad of 100 µg/mL. Cultures of microorganisms were added to the culture media. Control samples were NB and PDB media with a solution of the chemical preparation. The flasks were incubated in a 100 rpm shaking incubator. The experiments were conducted under a time (14 days) and temperature regime of 30 ± 2 °C for bacteria, and 28 ± 2 °C for fungi and a mixed culture of microorganisms. Samples for chemical analyses to determine penthiopyrad concentrations were periodically collected from the incubated flasks under sterile conditions. Additional samples for the evaluation of cellular metabolic activity were collected from the flasks in which penthiopyrad was degraded by *B. subtilis*. Pesticide residues were extracted from the samples taken for chemical analyses, and the obtained extracts were analyzed by gas chromatography (Agilent Technologies, Palo Alto, CA, USA) coupled with mass spectrometry. AlamarBlue reagent tests were used to assess cell metabolic activity.

#### 4.1.3. Evaluation of Bacteria *B. subtilis* Metabolic Activity

The metabolic activity of *B. subtilis* was examined with redox indicator AlamarBlue according to manufacturer’ protocol with some modification [52]. From each sample used for the assessment of cellular metabolic activity, 180 μL of the culture were collected under sterile conditions and placed in a black 96-well plate. 20 µL of AlamarBlue reagent (Thermo Fisher Scientific, Waltham, MA, USA) at a concentration of 0.01% were then added were added, followed by incubation in the dark for 10 min. Then fluorescence was measured with a Tecan INFINITE 200 Pro microplate reader (Tecan Group Ltd., Männedorf, Switzerland) at an excitation wavelength of 560 nm and an emission of 590 nm. The metabolic activity was expressed as relative fluorescence unit (RFU).

### 4.2. Field Trials

The supervised field trials were conducted in two commercial apple orchards, in two varieties: Gala (Józefów nad Wisłą; south-eastern Poland, Lublin voivodeship), and Golden Delicious (Rzeszów; south-eastern Poland, Podkarpackie voivodeship).

Standard agricultural practices, including pruning, fertilizing and soli management, were performed in the orchards during the growing seasons.

Apple trees were sprayed with Fontelis 200 SC (DuPont, Midland, MI, USA) (active substance: penthiopyrad, 200 g/L) at a dose of 0.75 L/ha [49]. In each test, fungicides were applied individually with a sprayer, Turbine N TNC 1000 (Annovi Reverberi, Modena, Italy) and Agrola 1500 (Zakład Handlowo-Produkcyjny AGROLA, Płatkownica, Poland) for Golden Delicious and Gala, respectively. The apple trees of Gala and Golden Delicious varieties were sprayed on August 16, 2017 and September 4, 2018, respectively, three weeks before harvesting of ripe apples. The biological preparation Zumba Plant^®^ (NaturalCrop, Warsaw, Poland) was applied 7 days after the treatment with the chemical preparation. Zumba Plant is an organic fertilizer enriched with strains of antagonistic microorganisms (Bacillus spp., Trichoderma spp.) and micorrhizal fungi (Glomus spp.). It stimulates the growth and development of plants and improves the fertility of the soil. This fertilizer also promotes a healthy growth of roots and the above ground parts of plants. The three strains of beneficial microorganisms contained in the fertilizer interact with each other. As a result, the product is characterized by its high efficiency, broad spectrum of activity, and ease of use [53].

Treatments with the chemical preparations were conducted in the entire experimental plot (ca. 0.5 ha) with a given variety, which was later divided into two blocks, I and II, each containing 6 rows. 7 days after the treatments with the chemical preparation, water was applied in block I (used as the control), and the treatment with biological preparation was performed in block II.

Weather conditions in Józefów were measured using the WatchDog 2900ET weather station (Spectrum Technologies, Inc., Aurora, IL, USA) installed in the orchard. Temperatures (°C) and rainfall (mm) were recorded from the date of first spraying to the end of the trial (Supplementary Figure S2A). Weather conditions in the Rzeszów region were taken from https://www.weatheronline.pl/ (Supplementary Figure S2B).

#### 4.2.1. Sampling

The first apple samples were collected about 12 h after treatment with the chemical preparation, and then every few days, for a total period of 21 days. Laboratory samples of fruit, weighing about 1.5 kg (10 units) each, were collected manually from randomly selected trees, one apple from one tree, in four replications [54]. On the first two sampling dates, 4 samples were taken from the entire experimental plot. After the treatment with the biological preparation, 4 apple samples of 10 units were collected from each block, where the control samples came from block I, and the test samples were picked from block II.

The fruit samples were transported to the laboratory in polyethylene foil bags, permanently and clearly marked. Laboratory samples were homogenized in a Blixter 4 (Robot Coupe, Vincennes, France), mixed to ensure representativeness, and then analytical samples of 10 g each were weighed. The samples were stored for a maximum of one week in a freezer at −17 °C in sealed 50 mL extraction tubes until the day of extraction.

#### 4.2.2. Determination of Penthiopyrad

##### Samples for Laboratory Experiments

Briefly, One hundred µL samples of liquid culture medium (NB and PDB) were transferred into polypropylene tubes, and 5 mL of acetone (Honeywell, Charlotte, NC, USA) and 0.5 g of anhydrous sodium sulfate (Chempur, Piekary Śląskie, Poland) were added. The contents of the tubes were shaken for 1 min (BenchMixerTM, Benchmark Scientific, Inc., Edison, NJ, USA) and then centrifuged at 3500 rpm for 5 min (MPW-350R, MPW MED. INSTRUMENTS, Warsaw, Poland). Then, 200 µL of acetone extract were transferred into a chromatographic vial, supplemented with 800 µL of petroleum ether (Chempur, Piekary Śląskie, Poland) and then 100 µL of internal standard (TPP, Triphenyl Phosphate, Supelco Inc., Bellefonte, PA., USA) of 21.6 µg/mL were added. The extracts were analyzed by gas chromatography with mass detection. The samples from laboratory tests were not stored before extraction. They were prepared and determined on the same day.

##### Samples for Field Experiments

The analysis of penthiopyrad in apples was conducted using the method based on the European Norm [55]. Briefly, 10 mL of acetonitrile were added to 50 mL polypropylene tubes containing 10 g of the analytical sample and shaken vigorously for 1 min (BenchMixerTM, Benchmark Scientific, Inc., Edison, NJ, USA). Buffer salts containing 4 g MgSO_4_ (Chempur, Piekary Śląskie, Poland), 1 g NaCl (Honeywell, Charlotte, NC, USA), 1 g sodium citrate (Chempur, Piekary Śląskie, Poland), and 0.5 g disodium citrate sesquihydrate (Sigma Aldrich, Saint Louis, MO, USA) were added and shaken vigorously again for 1 min (BenchMixerTM, Benchmark Scientific, Inc., Edison, NJ, USA). The contents of the tube were centrifuged at 3500 rpm (MPW-350R, MPW MED. INSTRUMENTS, Warsaw, Poland) for 5 min. Then, 5 mL of organic layer were transferred to a 15-mL polypropylene centrifuge tube containing clean-up sorbent consisting of 150 mg of PSA (Supelco Inc., Bellefonte, PA, USA) and 900 mg MgSO_4_ (Chempur, Piekary Śląskie, Poland). The contents of the tube were shaken for 1 min and centrifuged at 3500 rpm for 5 min. 750 µL of the purified extract were evaporated under a stream of nitrogen to dryness, then the dry residue was dissolved in 750 µL of petroleum ether, and 100 mL of TPP internal standard were added. The extracts were analyzed by gas chromatography with mass detection.

##### GC-MS Analysis of Penthiopyrad

The quantification of penthiopyrad was performed using the 7890A gas chromatograph (Agilent Technologies, Palo Alto, CA, USA) equipped with a mass detector, model 7000 (GC-MS/MS QQQ). The following analysis parameters were applied: injection of samples in the splitless mode, the injected volume—1 μL, carrier gas—helium (5.0 purity, flow 1 mL/min), and the ionization mode—electron (−70 eV), with temperatures of 250 °C for the transfer line, 230 °C for the ion source, and 150 °C for quadrupoles. The column temperature was maintained at 40 °C for 2 min, then increased to 300 °C at 30 °C/min, increased to 260 °C at 5 °C/min, and increased to 280 °C at 20 °C/min, and maintained for 8 min. Mass Hunter Software (version B.07.06, Agilent Technologies, Palo Alto, CA, USA, 2010) was used for instrument control and data acquisition and processing. Transitions of ions were monitored in the Multiple Reaction Monitoring (MRM) mode. In the qualitative analysis, monitored ion transitions were 302.1 → 151.9 (15) for penthiopyrad, and 326.1 → 128.0 (15) for the internal standard, while in the quantitative analysis they were 302.1 → 177.0 (15) and 326.1 → 228.4 (15) for penthiopyrad and the internal standard, respectively.

### 4.3. Determination of Mycotoxins in Apple Samples

The biological preparation used in the experiments contains fungi that can produce a number of metabolites, including mycotoxins [56]. Therefore, in each of the field experiments, apple samples were taken for mycotoxin determinations on the last sampling day, i.e., just before the fruit was deposited in the cold store. Samples were collected from rows treated with the biological preparation. The mycotoxin determination was conducted at the Mycotoxin Research Laboratory of the Faculty of Natural Sciences, the Kazimierz Wielki University in Bydgoszcz.

#### 4.3.1. Patulin

Five grams of the homogenized apple sample were shaken with 20 mL of acetonitrile:water (80:20, *v*/*v*) mixture for 60 min, and centrifuged at 5000 rpm for 10 min. Then, 8 mL of supernatant were passed through the push-through-type SPE column MycoSep 228 AflaPat (Romer, Newark, DE, USA). 4 mL of purified eluate and 20 µL of isotopic 13C-labeled PAT (13C-PAT; c = 50 µg/L) were transferred into a conical vial and evaporated to dryness under a gentle stream of nitrogen at 45 °C. The residue was redissolved in 1 mL of a mobile phase mixture of methanol:water (3:7, *v*/*v*), and then filtered on a 0.22 μm PTFE syringe filter.

Patulin detection was carried out using the Nexera high performance liquid chromatograph (HPLC) (Shimadzu, Tokyo, Japan) with a mass detector, 5500 QTrap (Sciex, Foster City, CA, USA). Mycotoxins were separated on a chromatographic column Gemini C18 (150 × 4.6 mm, 5 μm) (Phenomenex, Torrance, CA, USA). The chromatograph operating conditions are shown in Supplementary Table S3. The mass spectrometer operating conditions, and MRM transitions for patulin and the internal standard are shown in Supplementary Table S4.

#### 4.3.2. Trichothecenes and Zearalenone

A total of 12.5 g of the homogenized apple sample were shaken with 50 mL of acetonitrile:water (80:20, *v*/*v*) mixture for 60 min, and the extract was centrifuged at 5000 rpm for 10 min. The aliquot of 40 µL internal standard solution (13C-ZAN; c = 1000 µg/L) was added to 4 mL of the extract and the mixture was loaded onto the Bond Elut^®^Mycotoxin column (Agilent, Palo Alto, CA, USA). Then, 50 µL of the internal standard solutions (13C-DON; c = 2500 µg/L; 13C-T2; c = 250 µg/L and13C-HT2; c = 250 µg/L) were added to 2 mL of purified extract, and the mixture was evaporated to dryness using nitrogen at 45 °C. Then, 495 µL of MeOH:H_2_O (1:4, *v*/*v*) were added to the vial and the sample was vortexed.

Trichothecenes and zearalenone were determined using a high-performance liquid chromatography (HPLC; Shimadzu, Nexera, Japan) with mass spectrometry (API 4000, Sciex, Redwood City, CA, USA), and were separated on the Gemini-NX-C18 chromatographic column (150 × 4.6 mm, 3 µm; Phenomenex, Torrance, CA, USA). The chromatograph operating conditions are shown in Supplementary Table S5. The mass spectrometer operating conditions, and MRM transitions for trichothecenes and zearalenone and the internal standard are shown in Supplementary Table S6.

### 4.4. Method Validation

Validation was performed according to the European Union guideline SANTE [40]. The parameters of linearity (expressed as a coefficient of determination R^2^), trueness, and precision (expressed as an average recovery and a relative standard deviation, RSD) were assessed. The materials used for validation were apples and broth samples without penthiopyrad residues.

### 4.5. Statistical Analysis

Mean values (± SD) were calculated on a basis of at least three independent experiments. The statistical significance was assessed using Microsoft Office Excel with the Student’s *t*-test for independent samples with a two-trace distribution. The statistically significant differences were marked in the charts as: * *p* < 0.05; ** *p* < 0.01.

### 4.6. Estimation of Chronic and Acute Exposure

The consumer exposure to penthiopyrad residues in food products should be assessed in accordance with the Community procedures and practices, and taking into account recommendations published by the World Health Organization. On February 1, 2018, EFSA (The European Food Safety Authority) introduced a revised model for calculating the acute and chronic consumer exposure—PRIMo rev. 3. [57]. In this paper, the assessment of chronic (IEDI, as mg/kg bw/day) and acute (IESTI, as mg/kg bw/day) exposure for children and adults was undertaken, using the model recommended by EFSA PRIMo rev. 3. ADI (0.1 mg/kg bw) and ARfD (0.75 mg/kg bw) values for penthiopyrad are derived from a pesticide database [58].

## Figures and Tables

**Table 1:** Pentiopirad concentrations in control samples and after the application of *B. subtilis*, *T. harzianum*, and a mixed culture of *B. subtilis* and *T. harzianum*.

Days	Penthiopyrad + *B. subtilis* ± SD [µg/mL]	Penthiopyrad Control ± SD [µg/mL]	Penthiopyrad + *T. harzianum* ± SD [µg/mL]	Penthiopyrad Control ± SD [µg/mL]	Pentiopirad + *B. subtilis* + *T. harzianum* ± SD [µg/mL]	Penthiopyrad Control ± SD [µg/mL]
0	114.8 ± 5.7	114.6 ± 5.1	106.7 ± 5.4	106.0 ± 2.5	109.8 ± 4.3	110.6 ± 2.4
3	106.3 ± 3.2	112.5 ± 4.6	69.1 ± 7.6	105.0 ± 2.8	80.3 ± 2.1	105.3 ± 2.7
5	98.4 ± 4.8	105.3 ± 2.8	54.2 ± 3.8	101.8 ± 15.6	79.3 ± 7.1	109.3 ± 5.7
7	98.9 ± 6.9	104.6 ± 8.7	45.3 ± 3.2	101.4 ± 6.5	77.5 ± 8.8	105.1 ± 5.8
14	99.5 ± 3.5	104.0 ± 4.2	42.9 ± 7.6	99.4 ± 5.1	71.5 ± 3.5	100.8 ± 6.0

**Table 2:** Sampling dates and penthiopyrad residue levels after treatment with Fontelis 200 SC on 8/16/2017 (Gala variety) and 9/4/2018 (Golden Delicious variety), and with Zumba Plant^®^ on 8/23/2017 (Gala variety) and 9/11/2018 (Golden Delicious variety).

Sampling Date	Number of Days after Chemical Treatment	Penthiopyrad Concentration [mg/kg]	SD [mg/kg]	Pentiopirad Concentration after Treatment with the Biological Preparation [mg/kg]	SD [mg/kg]
Gala variety					
8/17/2017	1	0.161	0.034	–	–
8/21/2017	5	0.150	0.044	–	–
8/24/2017	8	0.105	0.036	0.102	0.018
8/28/2017	12	0.065	0.013	0.064	0.008
8/31/2017	15	0.041	0.013	0.033	0.014
9/6/2017	21	0.037	0.014	0.032	0.010
Golden delicious variety					
9/5/2018	1	0.421	0.046	–	–
9/9/2018	5	0.261	0.015	–	–
9/12/2018	8	0.269	0.039	0.265	0.042
9/16/2018	12	0.166	0.010	0.165	0.053
9/19/2018	15	0.105	0.011	0.083	0.018
9/25/2018	21	0.078	0.017	0.063	0.017

## Supplementary Materials

The following are available online. Figure S1: Bacterial cell viability during 14 days of the experiment. Figure S2: Temperature and precipitation in a period from 8/16/2017 to 9/6/2017 during the experiment in the Gala variety in Józefów nad Wisłą (**A**), and from 9/4/2018 to 9/25/2018 during the experiment in the Golden Delicious variety in Rzeszów (**B**). Table S1: Method validation parameters: correctness, precision, relative standard deviation, limits of quantification (LOQ); matrix: culture media (NB and PDB) and apples. Table S2: Validation parameters—linearity, matrix: culture media (NB and PDB) and apples. Table S3: Acquisition parameters of a liquid chromatograph with an ion trap mass detector (MRM mode) used for patulin determination. Table S4: MRM transitions for patulin and an internal standard, and mass spectrometer operating conditions. Table S5: Acquisition parameters of a liquid chromatograph with a mass detector (MRM mode) used for trichothecenes and zearalenone determinations. Table S6: MRM transitions for trichothecenes, zearalenone, and an internal standard, and mass spectrometer acquisition parameters.
