# Supplementary material for: Influence of Bacillus subtilis and Trichoderma harzianum on Penthiopyrad Degradation under Laboratory and Field Studies
Source: Molecules. 2020 Mar 20;25(6):1421. doi: 10.3390/molecules25061421 (PMC7145286; doi:10.3390/molecules25061421)
Supplement: Supplementary file 1 [file molecules-25-01421-s001.pdf]

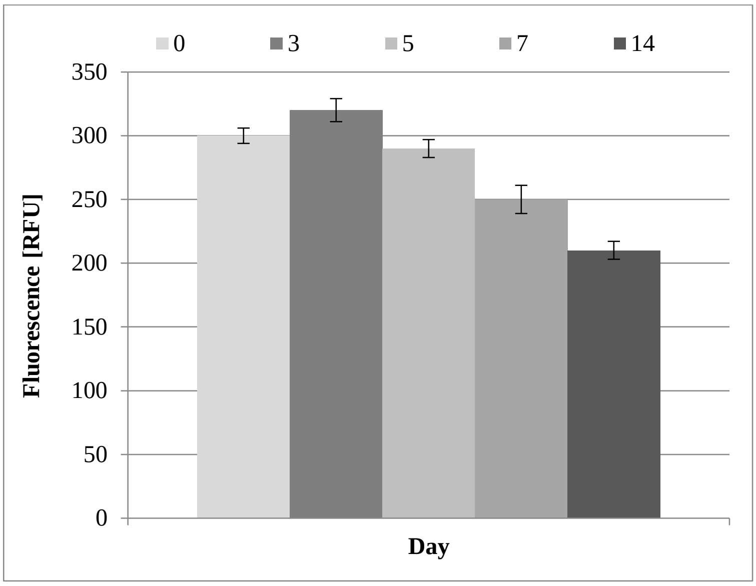

Figure S1. Bacterial cell viability during 14 days of the experiment

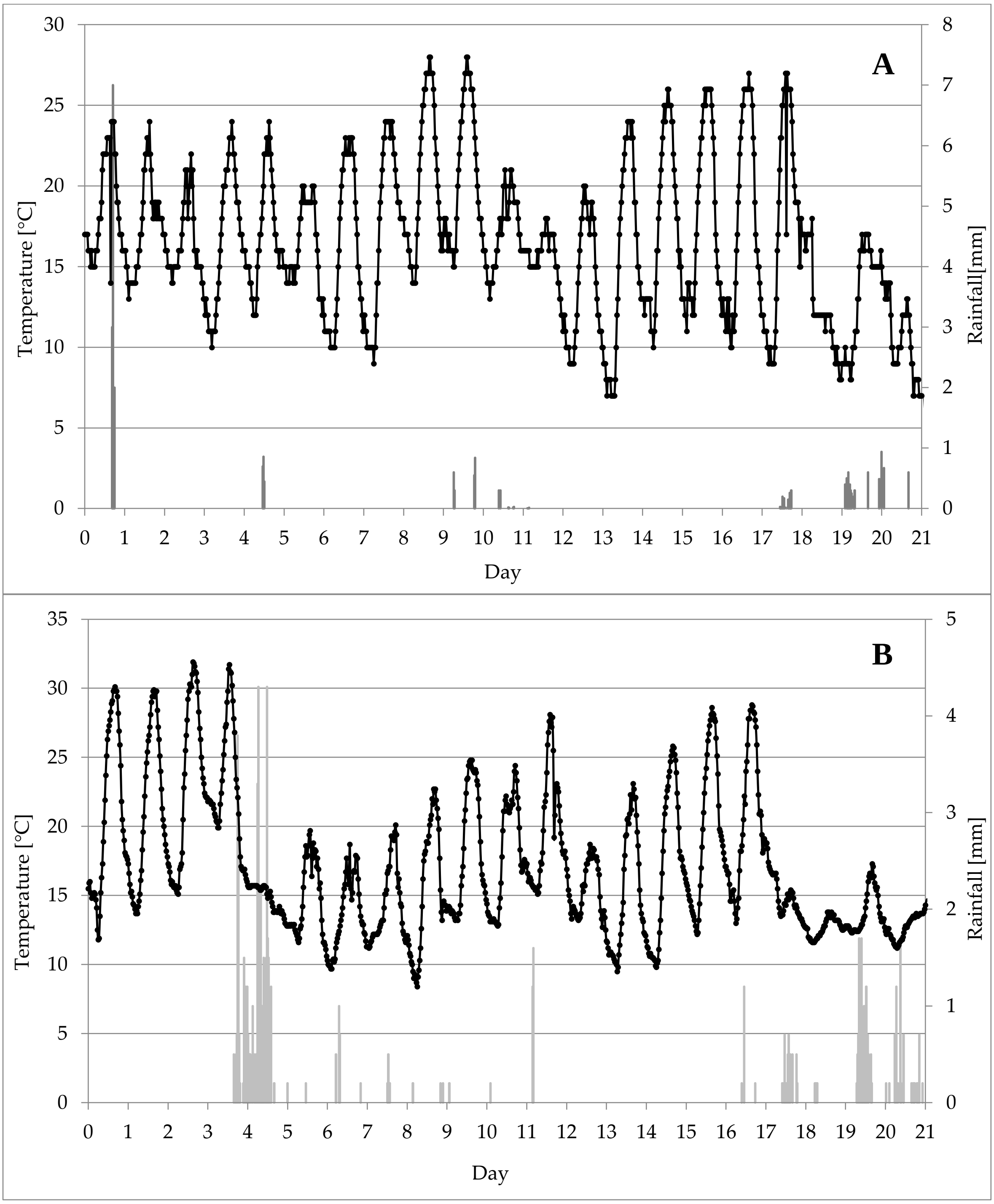

Figure S2. Temperature and precipitation in a period from 8/16/2017 to 9/6/2017 during the experiment in the Gala variety in Józefów nad Wisłą (A), and from 9/4/2018 to 9/25/2018 during the experiment in the Golden Delicious variety in Rzeszów (B)

Table S1. Method validation parameters: correctness, precision, relative standard deviation, limits of quantification (LOQ); matrix: culture media (BO and PDB) and apples

Active substance	Matrix	Fortification level [mg/kg]	Recovery [%]					Average recoveries [%]	SD [%]	RSD [%]	LOQ [mg/kg]
			I	II	III	IV	V				
Penthiopyrad	BO	1	97.5	105.0	107.0	105.5	102.5	103.5	3.7	3.6	1
		100	117.5	121.0	113.8	113.3	115.5	116.2	3.2	2.7	100
	PDB	1	89.0	88.5	109.5	98.5	102.0	97.5	8.9	9.2	1
		100	112.8	115.0	112.5	109.0	107.8	111.4	3.0	2.7	100
	Apples	0.01	118.0	118.5	117.0	112.5	110.0	115.2	3.7	3.3	0.01
		1	115.5	113.5	109.0	105.0	112.5	111.1	4.1	3.7	0.01

Table S2. Validation parameters - linearity, matrix: culture media (BO and PDB) and apples

Active substance	Matrix	No. of fortification levels	Linearity [mg/kg]	Calibration curve equation y = ax + b		Coefficients of determination
				a	b	R
Penthiopyrad	BO	5	0.004-0.4	348.9	20.8	0.9902
	PDB	5	0.004-0.4	443.2	28.2	0.9901
	Apples	5	0.01-1	32915.6	8.1	0.9983

Table S3. Acquisition parameters of a liquid chromatograph with an ion trap mass detector (MRM mode) used for patulin determination

**Flow rate**	1 mL/min
**Injection volume**	5 μL
**Mobile phases:**	
**A**	methanol/water/acetic acid 10/89/1 (v/v/v)
**B**	methanol/water/acetic acid 97/2/1 (v/v/v)
	(both phases contained 5 mmol/L ammonium acetate)
**Gradient**	0 % B for 2.0 min<br>50 % B from 2.0 to 5.0 min<br>100% B from 5.0 to 6.0 min<br>100 % B to 7.0 min, then<br>0 % B to 12 min
**Curtain gas**	30 psi
**Ionspray voltage / temperature**	-4500 V / 550°C
**Ion source gas 1**	60 psi
**Ion source gas 2**	60 psi

Table S4. MRM transitions for patulin and an internal standard, and mass spectrometer operating conditions

	Ionization	Precursor ion	Product ions	Declustering potential	Collision energy	Cell exit potential

		[m/z]	[m/z]	[V]	[V]	[V]
Patulin	$[M-H]^-$	153.0	109.1/81.0	-85	-12/-16	-7/-9
13C-Patulin	$[M-H]^-$	160.0	115.0/86.1	-95	-12/-16	-13/-13

Table S5. Acquisition parameters of a liquid chromatograph with a mass detector (MRM mode) used for trichothecenes and zearalenone determinations

**Flow rate**	0.5 mL/min
**Injection volume**	7 µL
**Mobile phases:**	
**A**	1% CH_3COOH in H_2O
**B**	methanol
	(both phases contained 5 mM CH_3COONH_4)
**Gradient**	30 % B for 0.5 min<br>90 % B from 0.5 to 6.0 min<br>90 % B to 10.0 min, then<br>30 % B to 15 min
**Curtain gas**	25 psi
**Collision gas**	6 psi
**Ionspray voltage /temperature**	-4000 V (negative polarity); 5000 V (positive polarity) / 500°C
**Ion source gas 1**	50 psi
**Ion source gas 2**	50 psi

Table S6. MRM transitions for trichothecenes, zearalenone, and an internal standard, and mass spectrometer acquisition parameters

	**Ionization**	**Precursor ion [m/z]**	**Product ions [m/z]**	**Declustering potential [V]**	**Collision energy [V]**	**Cell exit potential [V]**
13C-Deoxynivalenol	$[M+Ac]^-$	370.2	310.0	-50	-14	-7
13C-HT-2 Toxin	$[M+NH_4]^+$	464.1	278.1	51	17	18
13C-T-2 Toxin	$[M+NH_4]^+$	508.3	322.1	61	19	8
13C-Zearalenone	$[M-H]^-$	335.1	139.9	-100	-42	-7
Deoxynivalenol	$[M+Ac]^-$	355.1	264.8/58.9	-35	-20/-38	-17/-1
Diacetoxyscirpenol	$[M+Ac]^-$	384.1	307.0/247.0	51	17/19	20/16
HT-2 Toxin	$[M+NH_4]^+$	442.2	215.0/263.0	51	19/17	14/18
Nivalenol	$[M+Ac]^-$	371.1	281.0/59.0	-40	-22/-40	-14/-5
T-2 Toxin	$[M+NH_4]^+$	484.1	215.0/185.0	61	25/29	14/12
Zearalenone	$[M-H]^-$	317.1	130.8/174.9	-85	-40/-32	-7/-9
